# Evaluating the Whitening and Microstructural Effects of a Novel Whitening Strip on Porcelain and Composite Dental Materials

**DOI:** 10.4172/2161-1122.1000448

**Published:** 2017-08-29

**Authors:** Thair Takesh, Anik Sargsyan, Matthew Lee, Afarin Anbarani, Jessica Ho, Petra Wilder-Smith

**Affiliations:** Beckman Laser Institute, University of California, Irvine, CA

**Keywords:** Dental bleaching, Tooth whitening, Resin composite, Porcelain, Roughness, SEM, Profilometry

## Abstract

**Aims:**

The aim of this project was to evaluate the effects of 2 different whitening strips on color, microstructure and roughness of tea stained porcelain and composite surfaces.

**Methods:**

54 porcelain and 72 composite chips served as samples for timed application of over-the-counter (OTC) test or control dental whitening strips. Chips were divided randomly into three groups of 18 porcelain and 24 composite chips each. Of these groups, 1 porcelain and 1 composite set served as controls. The remaining 2 groups were randomized to treatment with either Oral Essentials^®^ Whitening Strips or Crest^®^ 3D White Whitestrips™. Sample surface structure was examined by light microscopy, profilometry and Scanning Electron Microscopy (SEM). Additionally, a reflectance spectrophotometer was used to assess color changes in the porcelain and composite samples over 24 hours of whitening. Data points were analyzed at each time point using ANOVA.

**Results:**

In the light microscopy and SEM images, no discrete physical defects were observed in any of the samples at any time points. However, high-resolution SEM images showed an appearance of increased surface roughness in all composite samples. Using profilometry, significantly increased post-whitening roughness was documented in the composite samples exposed to the control bleaching strips. Composite samples underwent a significant and equivalent shift in color following exposure to Crest^®^ 3D White Whitestrips™ and Oral Essentials^®^ Whitening Strips.

**Conclusions:**

A novel commercial tooth whitening strip demonstrated a comparable beaching effect to a widely used OTC whitening strip. Neither whitening strip caused physical defects in the sample surfaces. However, the control strip caused roughening of the composite samples whereas the test strip did not.

## Introduction

Home dental bleaching techniques were first introduced in the 1990s [1,2], and have become a common self-administered procedure due to their immediate and notable efficacy as well as their low cost. Dental bleaching agents often contain hydrogen or carbamide peroxide, whose effective lightening action is offset by reports of adverse effects on tooth structure and dental materials [3], especially when they are used repeatedly at high concentrations or applied over long periods of time. Reported adverse effects include softening, roughness, cracks and unsightly color changes [4]. Because composite and porcelain materials are widely used in dentistry [5], it is important to identify the effects of home bleaching on their integrity, longevity and appearance.

Several studies have evaluated the effects of bleaching agents on dental restorative materials. The results of some studies indicate that low concentrations of home-bleaching gel containing carbamide peroxide (10–16%) do not cause any significant changes on the surfaces of composite and porcelain restorative materials [6–9]. However, other researchers have reported bleaching-induced increased surface roughness of composite and porcelain when measured by Scanning Electron Microscopy (SEM) and roughness testing [10–13]. One study associated bleaching-induced loss of silicon and potassium dioxide with increased surface roughness in feldspathic porcelain [14]. Other investigators reported a reduction in composite material hardness after bleaching [10,15].

Hardness is defined as the resistance of a material against permanent surface penetration [9]. Reduced hardness and increased roughness may result from erosion of the organic matrix of the surface of dental materials exposed to whitening agents such as strips, gels or rinses [16]. Such damage can reduce material durability, facilitating the development of pitting, cracks and erosion in crowns and fillings [7]. Moreover, roughness facilitates plaque aggregation and bacterial growth ultimately increasing the risk of gingival and periodontal diseases [17]. Finally, bleaching-induced color shifts, especially those that cause a mismatch with surrounding tooth color, are esthetically undesirable [18].

Thus there exists a drive towards new dental whitening formulas that will reduce the adverse effects of existing formulations on the structure and color of teeth and restorative materials. The aim of this study was to investigate the effects of a novel bleaching strip formulation that is derived from natural materials on the surfaces and color of composite and porcelain dental restorative materials.

## Materials and Methods

### Overview

**Figure 1:**
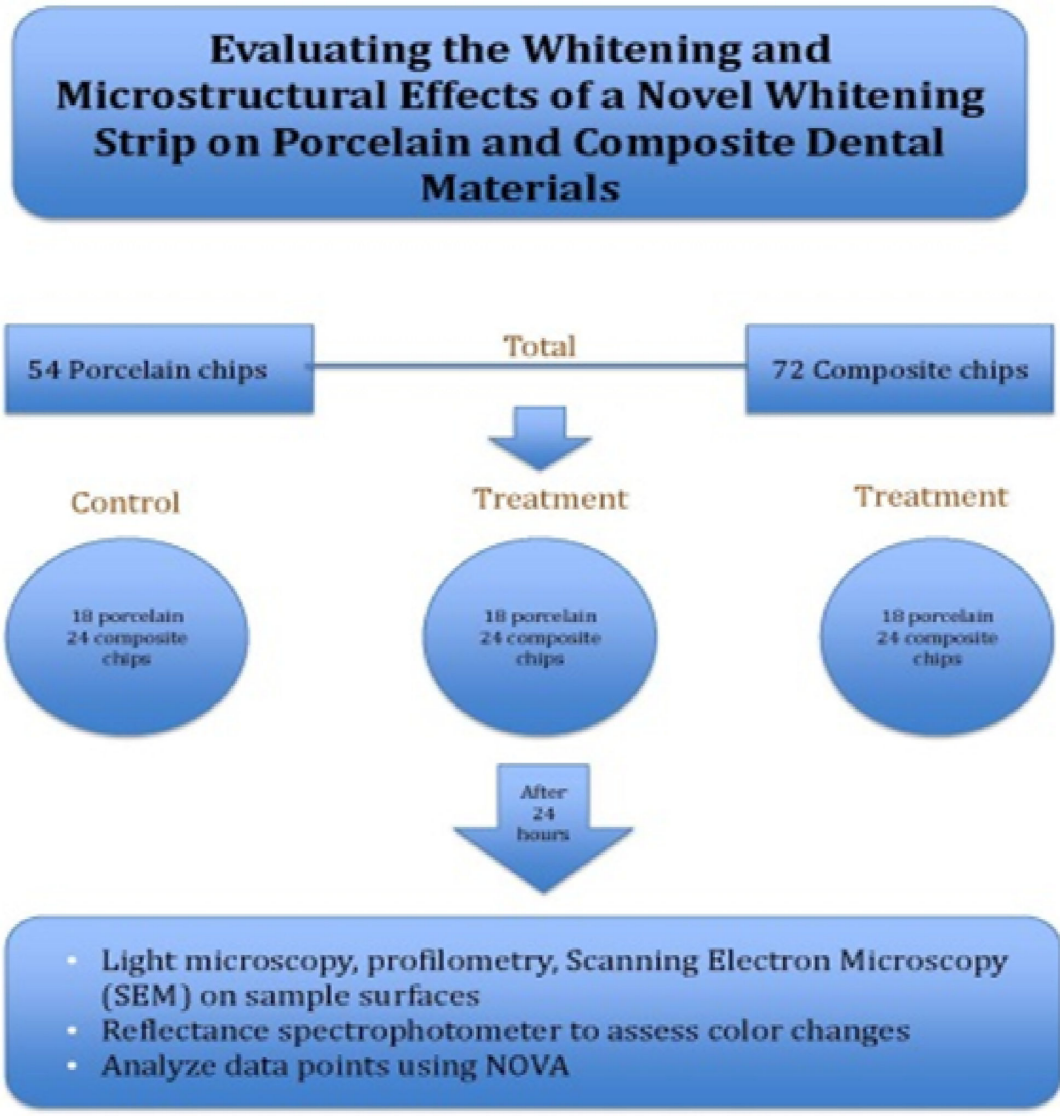
Evaluating the whitening and microstructural effects of a novel whitening strip on porcelain and composite dental materials.

A total of 54 porcelain chips from 6 different brands and 72 composite chip samples from 8 different brands were included in this study. Of these, 18 porcelain and 24 composite chips served as baseline untreated samples, while 36 porcelain chips and 48 composite chips served as active samples, undergoing timed application of a test or control whitening strip (Figure 1).

### Samples

Three rectangular-shaped chips (size 3 mm × 2 mm × 1 mm) were prepared from each composite and porcelain sample block. These blocks had been classified as defect-free by an experienced dentist (28 years of dental practice) using a loupe and headlamp. All chips were stored in a 0.5% chloramine-T solution for 48 hours prior to start of the study, then stained by overnight immersion in a concentrated black tea solution produced by steeping 2 teabags (Lipton^®^ yellow label black tea) in ½ cup of boiling water for 2 hours. One of each chip “triplet” was held back as untreated baseline sample, then these 18 porcelain and 24 composite baseline samples were stored in distilled water in a sealed and labeled double-walled container at temperature of 4°C and 100% humidity, and protected from ambient light. The 36 porcelain and 48 composite test chips were divided into two random groups, mounted on to a strip to avoid handling the samples directly and also stored in distilled water as described above. The active intervention chips were randomized to treatment either with the test (Oral Essentials^®^, Beverly Hills, CA 90210) or the control whitening strips (3D Crest^®^, Procter & Gamble, Cincinnati, OH 45202 USA) (Table 1).

The following materials were tested:

**Composites:** Venus Diamond Composite A1 (HerauesKulzer, South Bend, IN 46614, USA)
Esthelite quick composite A1 (Tokuyama Dental America, Encinitas, CA 92024, USA)
EsthetX HD composite A1 (Dentsply, York, PA 17401, USA)
Tetric Flow composite A1 (Ivoclar, Amherst, NY 14228, USA)
Clearfil Majesty composite A1 (Kuraray Noritake Dental, 65795 Hattersheim am Main, Germany)
Herculite Ultra A1 (Kerr, Orange, CA 92867, USA)
Ultra Composite A1 (3M Espe, Irvine, CA 92614, USA)
Tetric EVO flow Composite A1 (Ivoclar Vivadent, Amherst, NY 14228, USA)
**Porcelains:** Emax A1 (Glidewell Dental Laboratories, Newport Beach, CA 92660, USA)
Creation A1 (Jensen Dental Laboratories North Haven, CT 06473, USA)
Noritake Zirconia CZR A1 (Kuraray Noritake Dental, 65795 Hattersheim am Main, Germany)
Ceramco 3 A1 (Dentsply, York, PA 17401, USA)
Vita A1 (Vita, Yorba Linda, CA 92887, USA)
Noritake EX-3 A1 (Kuraray Noritake Dental, 65795 Hattersheim am Main, Germany)
Emax A1 (Glidewell Dental Laboratories, Newport Beach, CA 92660, USA)
Creation A1 (Jensen Dental Laboratories North Haven, CT 06473, USA)
Noritake Zirconia CZR A1 (Kuraray Noritake Dental, 65795 Hattersheim am Main, Germany)
Ceramco 3 A1 (Dentsply, York, PA 17401, USA)
Vita A1 (Vita, Yorba Linda, CA 92887, USA)
Noritake EX-3 A1 (Kuraray Noritake Dental, 65795 Hattersheim am Main, Germany)

### Primary end points

Data were collected at the following time points:

0: baseline, 1 hr, 3 hr, 6 hr, 9 hr, 12 hrs, 18 hrs, 24 hrs whitening exposure.
Data points collected for each time point included:
  1. Microscopy to monitor the surface structure
  2. Colorimeter measurement
  3. Photograph under same lighting condition
Data points collected at 24 h only included
  1. SEM
  2. Profilometry

### Data collection

#### Light microscopy

An Olympus Cap Optical SZX Light Microscope was used to acquire images of a total 126 samples in the three groups. Three different magnifications images (X10, X25 and X35) were used to identify any cracks, pits or fissures on the surface of the samples. The untreated group was used as a baseline for comparison.

#### Scanning electron microscopy

A total of 27 porcelain and 36 composite chips were air dried and prepared on Aluminum specimens mount disks using colloidal silver liquid (Ted Pella, CA, USA) and coated with 9.31 nm thick gold alloy. A Magellan 400L SEM was used to analyze the samples under high vacuum conditions. Three different images were taken from specific areas of the surfaces of samples with three magnifications (500, 1000 and 3000). Treated areas were compared to untreated baseline samples by a blinded pre-trained, pre-standardized scorer. Differences were scored as mild, moderate and severe.

#### Colorimetry

The L* and b* color values of each sample were recorded under carefully standardized lighting conditions using a reflectance spectrophotometer Colorimeter (PCE-CSM 1, PCE Instruments™, PCE Instrumentation, Alicante, Spain).

#### Profilometry

A profilometer (Perthometer M2, Mahr GmbH; Göttingen, Germany) was used for measuring surface roughness. The diameter of the tip of the profilometer was 2.4 mm, and the accuracy was 0.5 mm/sec. Measuring path was set to 5.5 mm. Five measurements were performed with a cut off value of 250 µm. The average surface roughness of each specimen was recorded. The profilometer was also calibrated after every seventh specimen measurement. Measurements were conducted on the center of each specimen.

### Data analysis

Colorimetry results were analyzed using repeated measures of variance technique (α=0.05) followed by Bonferroni post-test Surface roughness results were evaluated by repeated measures of variance technique (α=0.05). Coincidences were eliminated by Duncan test.

## Results

### Light microscopy

**Figure 2:**
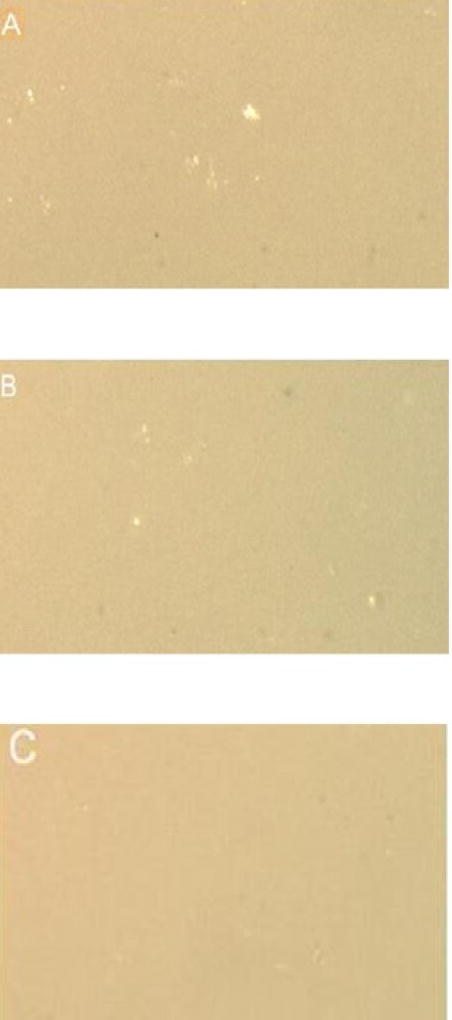
Composite surface after 24 h exposures to: A: Oral Essentials^®^ Whitening Strips; B: Crest® 3D White Whitestrips™; C: Untreated chips. Magnification X35, light microscopy.

**Figure 3:**
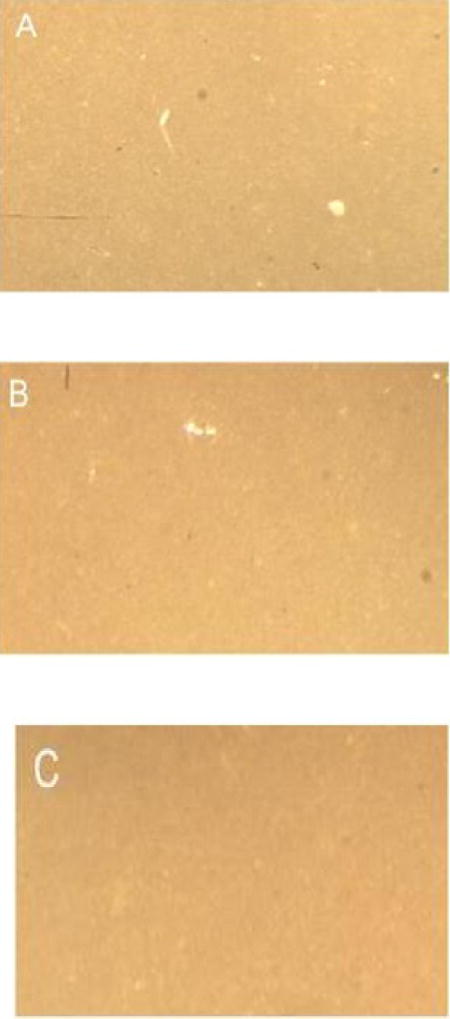
Porcelain surface after 24 h exposures to: A: Oral Essentials^®^ Whitening Strips; B: Crest® 3D White Whitestrips™;; C: Untreated chips. Magnification X35. Light microscopy.

No surface damage and no differences in surface integrity between untreated and bleached chips were observed on any of the sample surfaces, regardless of color, material and exposure duration. Representative photographs are shown in Figure 2A–2C (Composite samples) and Figure 3A–3C (Porcelain samples).

### Scanning Electron Microscopy

**Figure 4:**
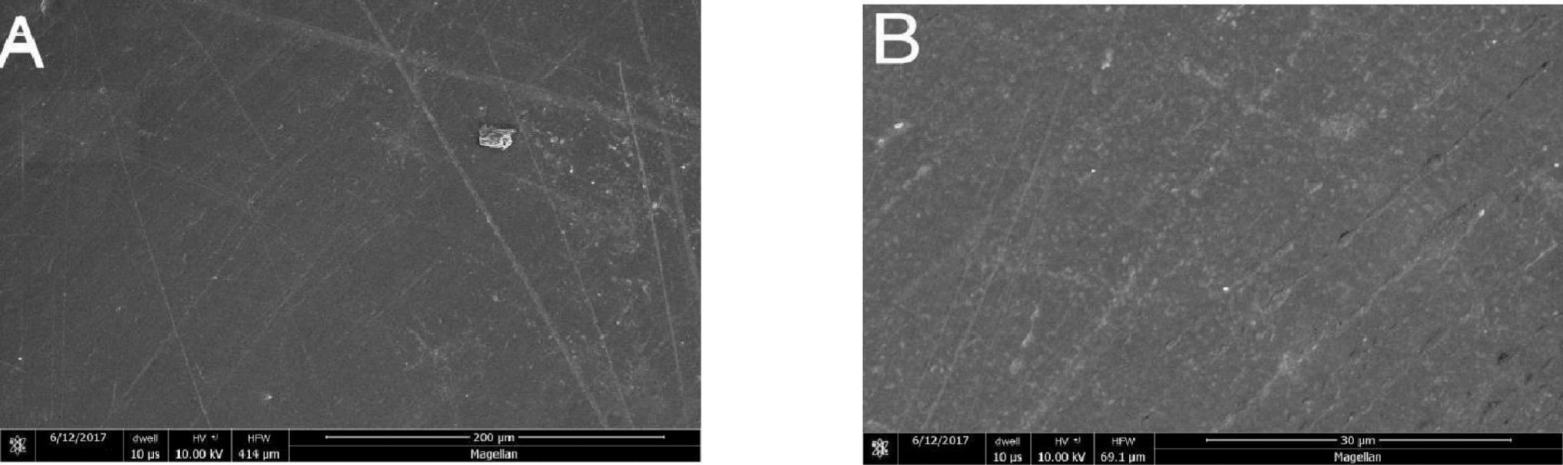
Composite surface after 24 h exposure to Oral Essentials^®^ Whitening Strips. A: X500; B: X3000. SEM.

**Figure 5:**
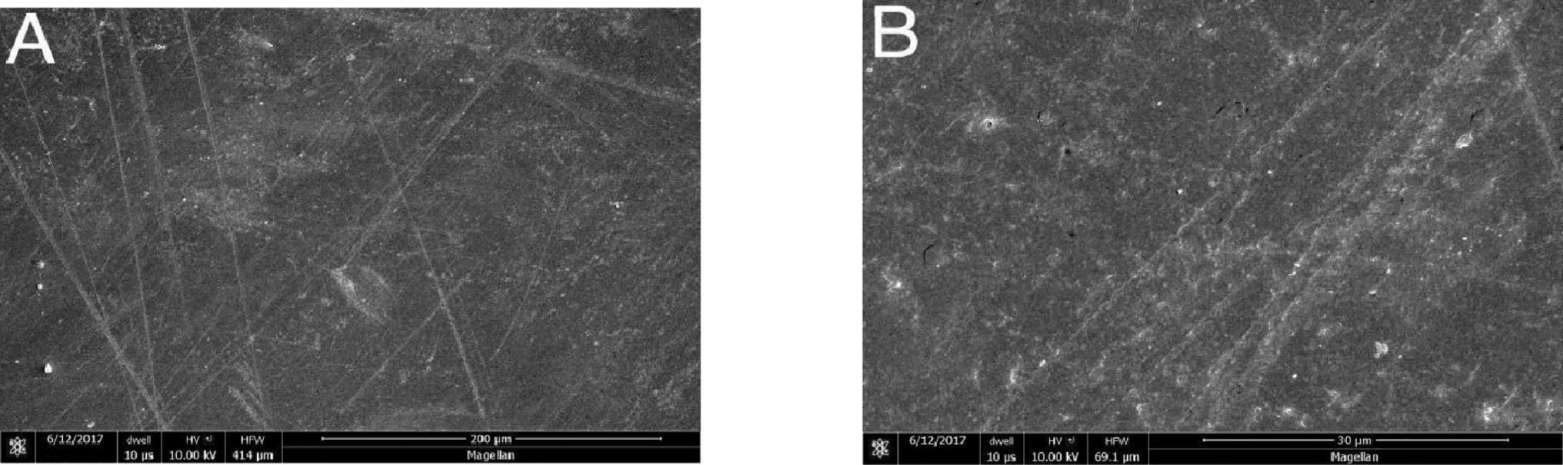
Composite surface after 24 h exposure to Crest® 3D White Whitestrips™. A: X500; B: X3000. SEM.

**Figure 6:**
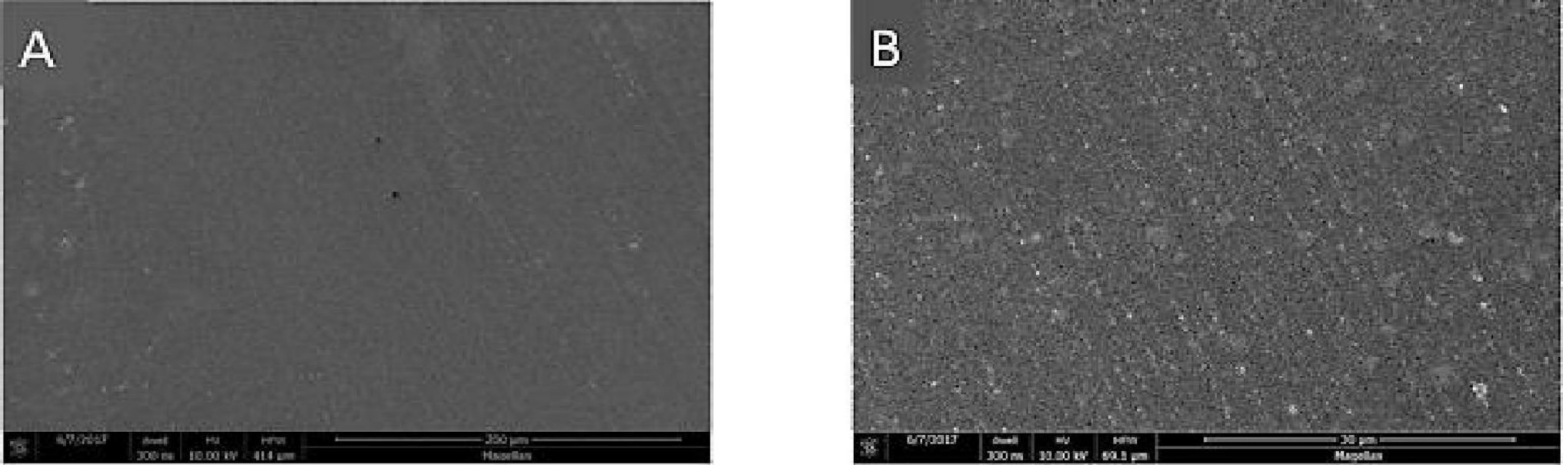
Composite surface for untreated chips. A: X500; B: X3000. SEM.

**Figure 7:**
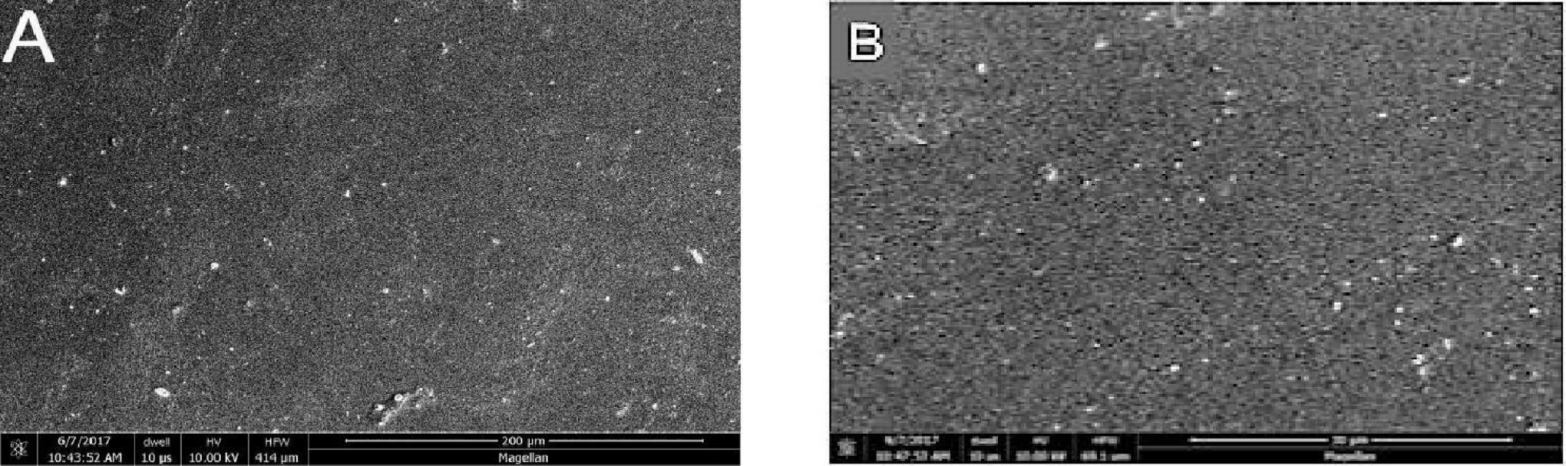
Porcelain surface after 24 h exposure to Oral Essentials^®^ Whitening Strips. A: X500; B: X3000. SEM.

**Figure 8:**
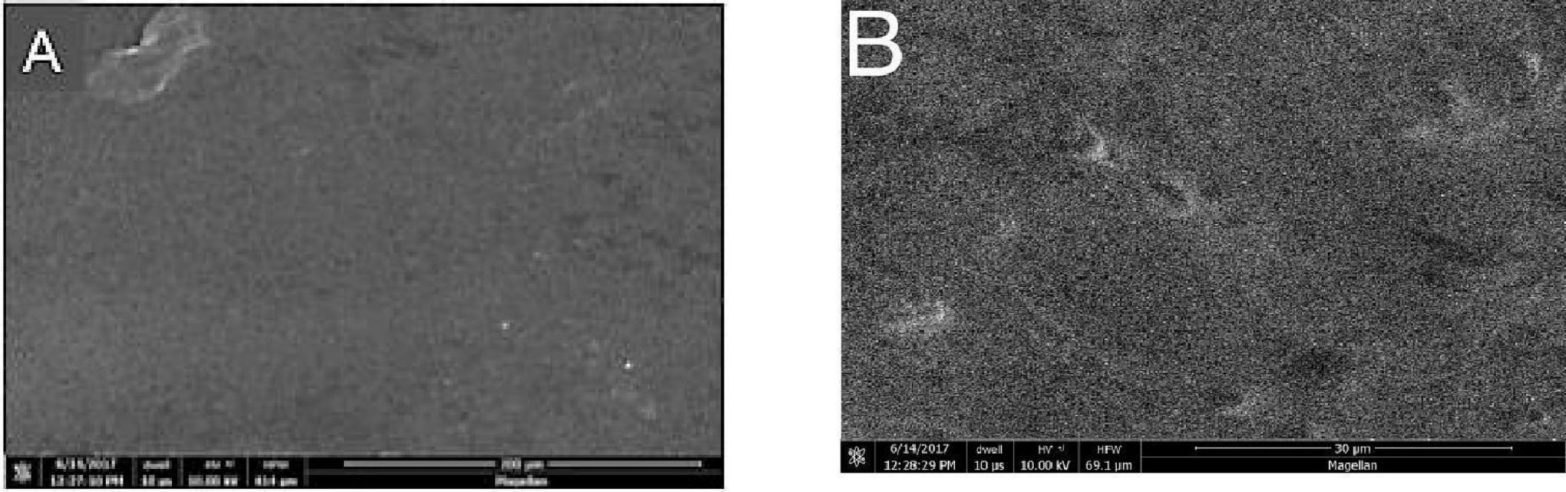
Porcelain surface after 24 h exposure to Crest® 3D White Whitestrips™. A: X500; B: X3000. SEM.

**Figure 9:**
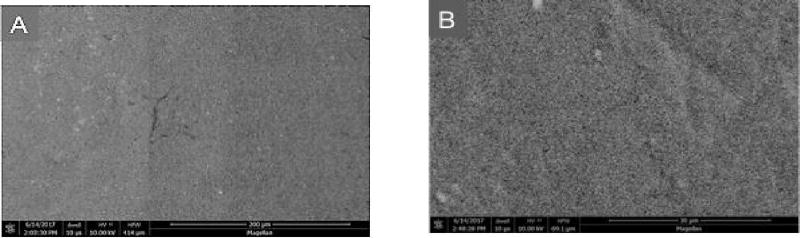
Porcelain surface for untreated chips. A: X500; B: X3000. SEM.

**Figure 10:**
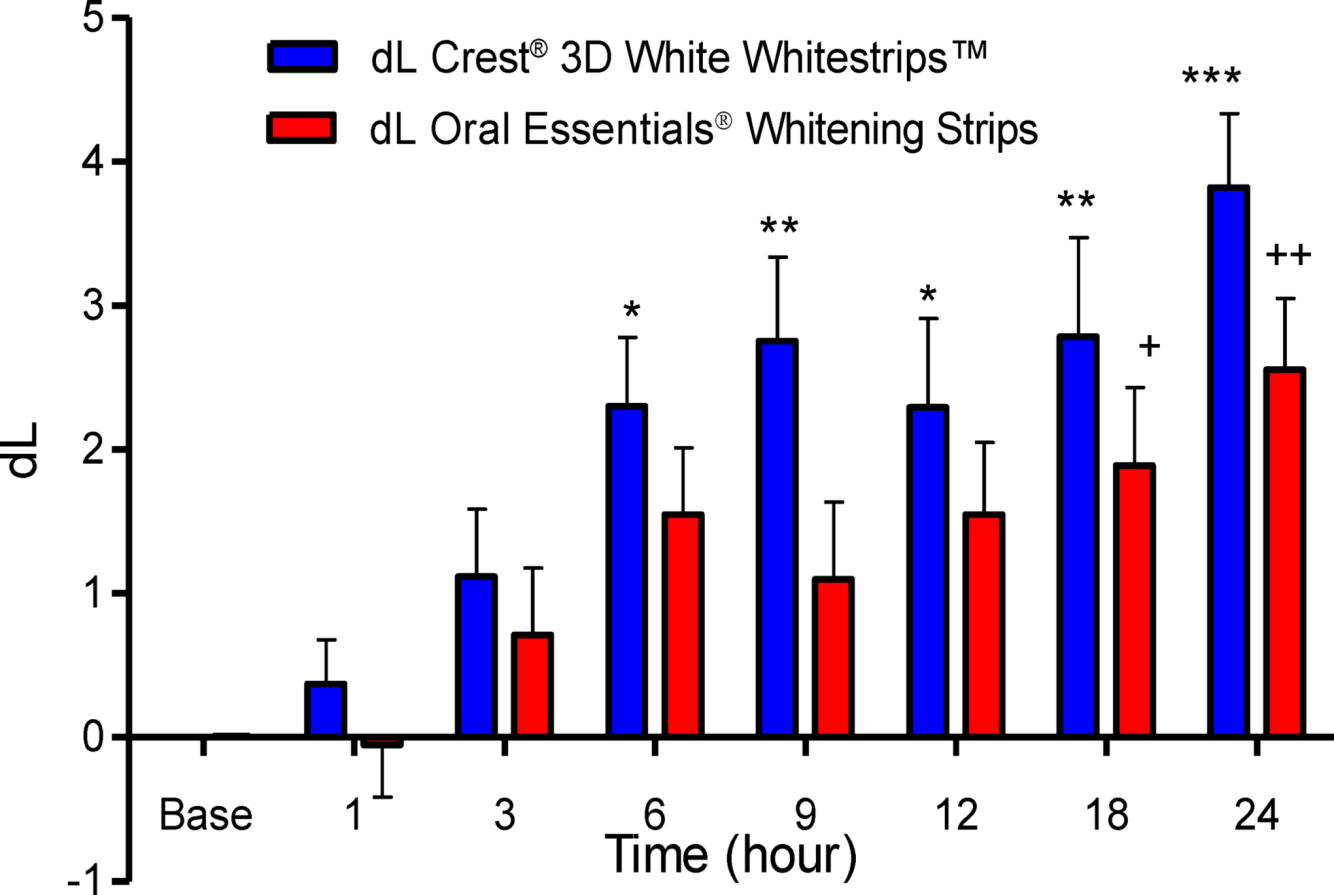
Effects of each whitening strip on composite dL values. Data are presented as mean ± SEM; n=8. Mean dL value for Composite after sequential hour-long treatment with bleaching strips.

At higher magnifications, greater surface roughening was consistently observed in composite and porcelain samples exposed to the control strips than those treated with the test strips. Representative photomicrographs are shown in Figures 4–6 (A and B) for Composite samples and Figures 7–10 (A and B) for Porcelain samples.

### Colorimetry

#### Composite Samples

**Figure 11:**
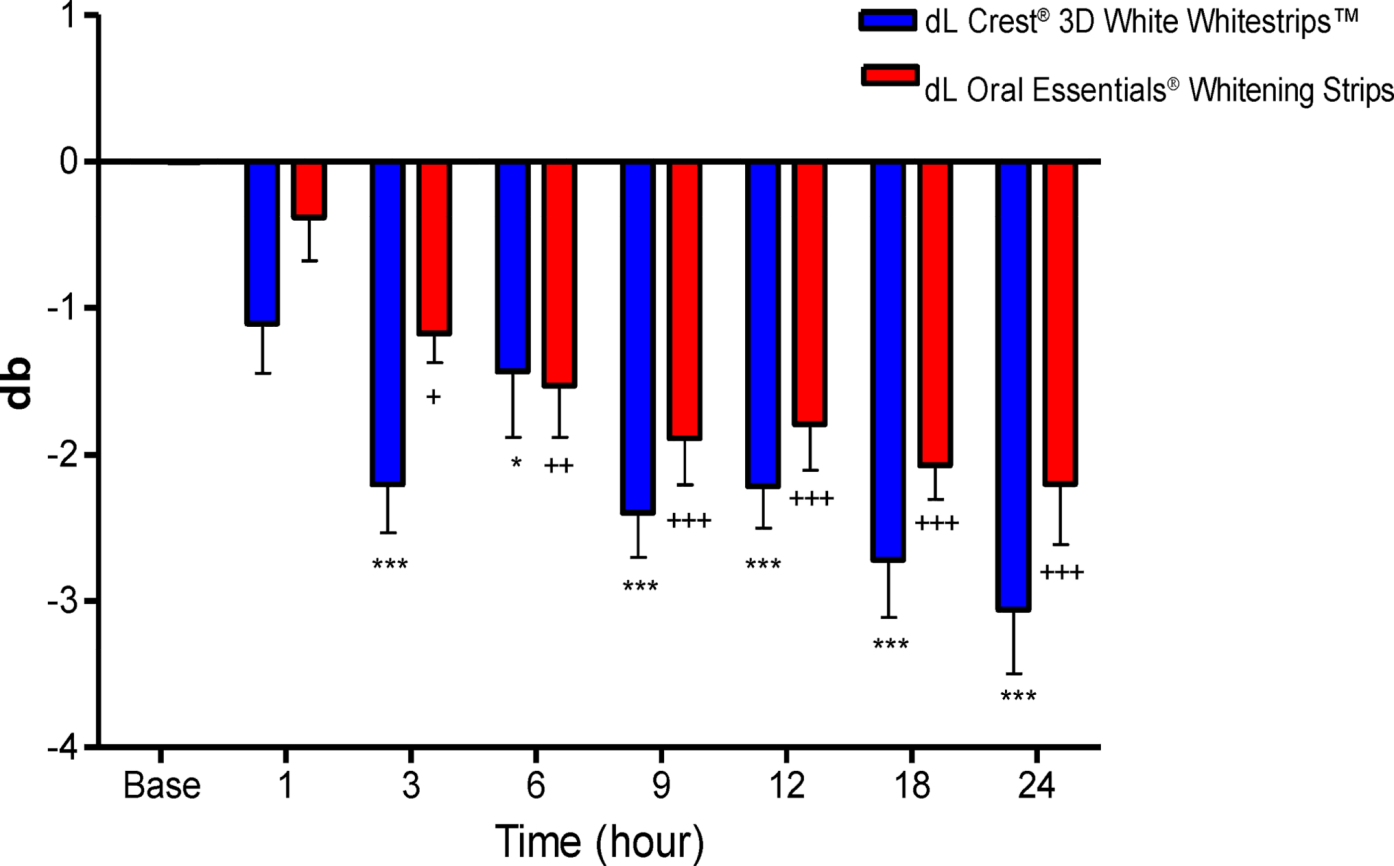
Effects of each whitening strip on composite db values. Data are presented as mean ± SEM; n=8. Mean db value for Composite after sequential hour-long treatment with bleaching strips.

L and b color values of the composite samples underwent progressive increases that paralleled the duration of whitening strip exposure. Mean color changes at each time point are shown in Tables 2 and 3 and Figures 10 and 11. The control strip caused significant increases in dL values after 6, 9, 12, 18, and 24 hour exposure times [one way ANOVA, (F_7,63_=6.5, *P*<0.0001)], and in db values after 3, 6, 9, 12, 18 and 24 hour exposure times [one way ANOVA, (F_7,63_= 8.28, *P*<0.0001)]. The test strip caused significant increases in dL values after 18 and 24 hour exposure times [one way ANOVA, (F_7,63_=4.01, *P*<0.0001)], and in db values after 3, 6, 9, 12, 18 and 24 hour exposure times [one way ANOVA, (F_7,63_=7.64, *P*<0.0001)]. There was no difference in bleaching effects between the control and the test treatment at any time point [two way ANOVA followed by Bonferroni post-test P>0.05], thus the 2 treatments showed equivalency between each other at all-time points.

#### Porcelain samples

**Figure 12:**
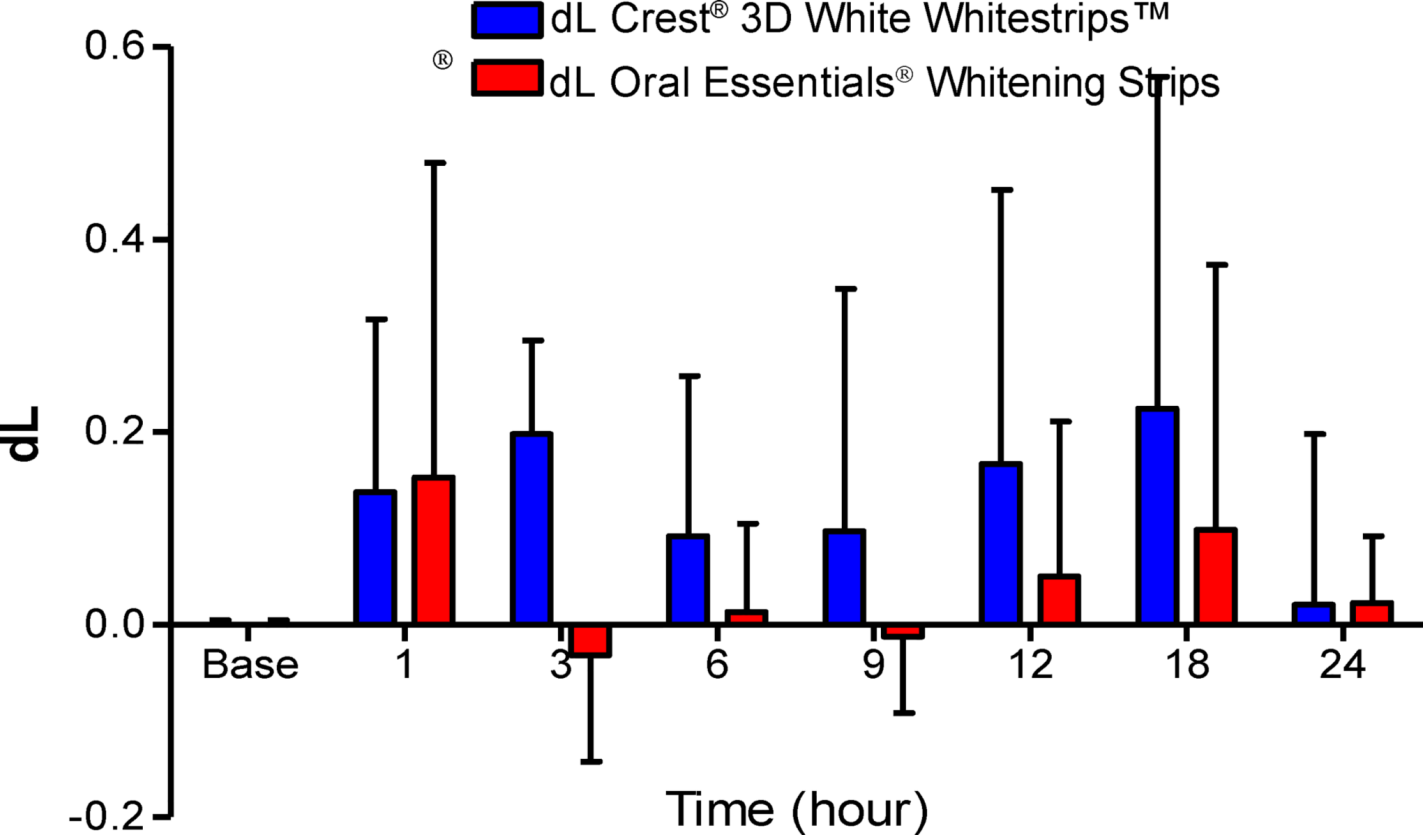
Effects of each whitening strip on Porcelain dL values. Data are presented as mean ±SEM; n=6. Mean dL value for Porcelain after sequential hour-long treatment with bleaching strips.

**Figure 13:**
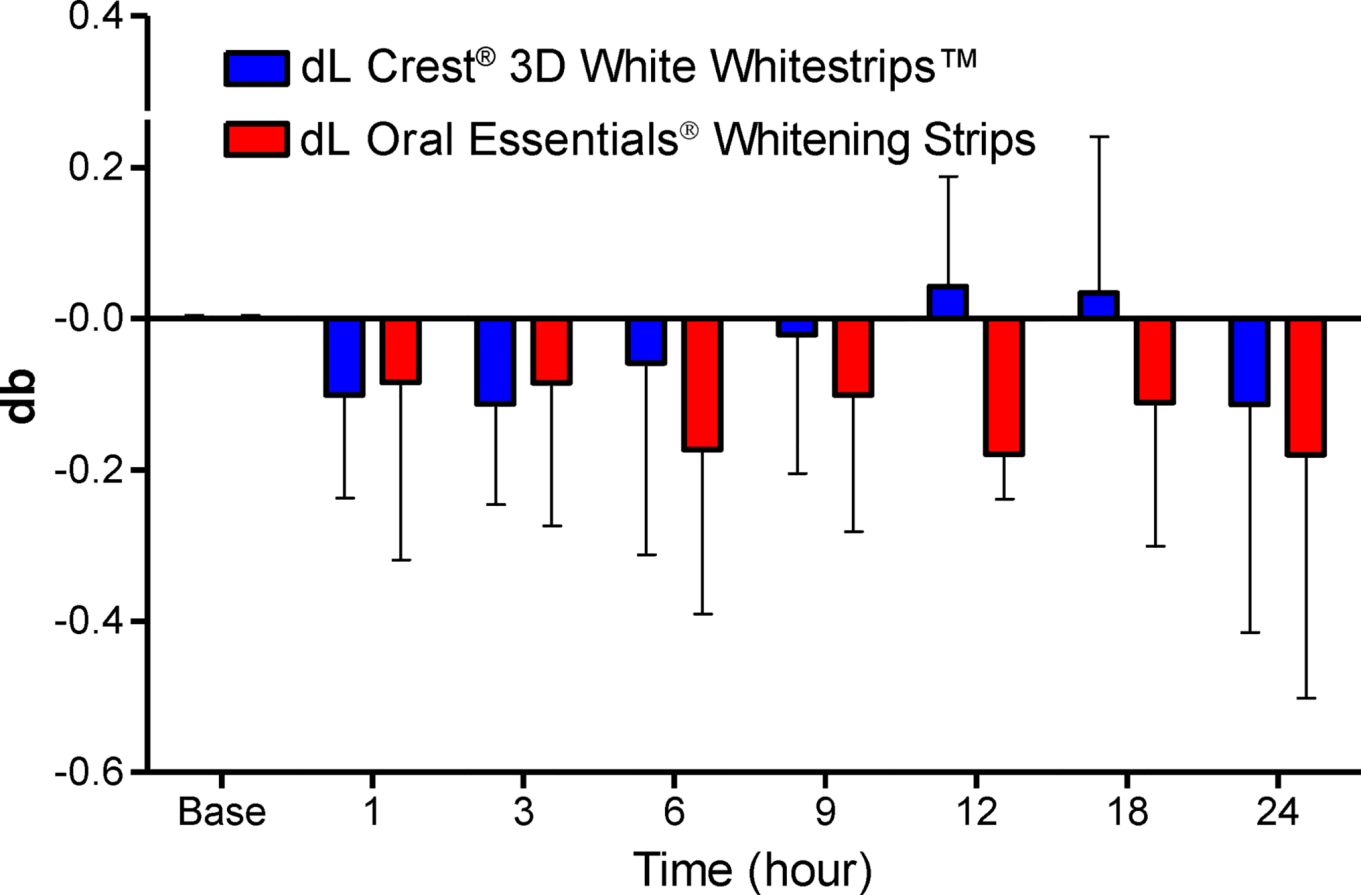
Effects of each whitening strip on Porcelain db values. Data are presented as mean ± SEM; n=6. Mean db value for Porcelain after sequential hour-long treatment with bleaching strips.

L and b values for the porcelain samples underwent only minimal change over time. Mean color values at each time point are shown in Tables 4 and 5 and Figures 12 and 13. No significant effect on dL and db values was observed resulting from exposure to either category of whitening strip. Moreover, no significant differences in dL and db values after use of either strip were determined (two way ANOVA P>0.05.)

### Profilometry

Significantly increased post-whitening roughness was documented in the composite samples exposed to the control bleaching strips (ANOVA, P>0.05). No such changes were observed in the porcelain samples after either whitening treatment.

## Discussion

A novel dental whitening strip for in-home use had comparable bleaching effects as a widely used control strip. While neither strip had any effects on composite or porcelain surfaces that were discernible to the naked eye or using light microscopy, the control strip caused surface roughening of all composite surfaces as evaluated using SEM and profilometry.

The effects of bleaching on the most commonly used dental materials, and especially on composite and porcelain, have been studied extensively. While some previous studies reported no change in composite and porcelain surface after bleaching [6,19–22], other investigators have found significant surface roughening in various resin and porcelain materials after exposure to hydrogen or carbamide peroxide [10,23,24]. In another study, bleaching agents were shown to decrease porcelain surface hardness without affecting its roughness [10,19].

Factors that may contribute to the wide range of results reported in the literature may be due to variations in the preparation and finishing of test samples, different study designs [20], and wide-ranging formulations of the bleaching materials used in these studies [25]. For example, one study found that the level of surface polish may affect composite susceptibility to surface damage from bleaching. The investigators reported no significant change in the surface roughness of well finished composite samples after bleaching, whereas the surfaces of unpolished resin samples became significantly rougher after the application of a whitening formulation [26]. *Ex vivo* studies may produce different results than *in vivo* investigations due to the very different environmental conditions in the laboratory vs. the oral cavity. In laboratory studies, samples surfaces often are not consistently wet and the temperature is usually lower than inside the mouth (20°C compared to 37°C) [27]. These two factors will tend to enhance the adverse effects of whitening agents within the oral cavity as compared to *ex vivo* models.

In this study, a colorimeter was used to measure color changes in all samples according to the standard CIELAB COLOR protocol. Both bleaching agents produced minimal color change over time on the different porcelain chips that were used in this study. This is most likely due to several factors: [1] the staining protocol was very moderate, producing little measurable staining in the porcelain samples and [2] the bleaching protocol was conservative. This finding is positive in that it confirms that the whitening strips used in this study will not alter the color of porcelain restorations, with no negative effects on finely tuned color matches between teeth and porcelain restorations. However, significant whitening effects were observed in all composite samples after 9 and 18 hours of sequential exposure to either strip. These results are consistent with those reported by Monaghan et al. [28], who determined significant color changes in composite restorations after in-office bleaching procedures. Other studies reported similar findings in composite materials [29,30]. The oxidation of stains combined with micro-structural surface changes on the surface of resin materials may explain the color changes caused by whitening formulations on composite chips [21]. Conversely, investigators found no evidence of color change in composite materials after applying a 35% hydrogen peroxide formulation for 30 minutes to their surfaces [31]. Thus, in-home bleaching must be performed carefully if composite restorations are present, to ensure that color matches between the teeth and composite restorations remain balanced throughout dental whitening procedures. Moreover the appearance of stained composite restorations may be improved if care is taken to monitor and match whitening effects on composites and adjacent tooth structures.

## Conclusion

The surface roughening observed in composite samples after exposure to a common OTC dental whitening strip was not observed in such samples after exposure to a novel whitening strip formulation. Both strips caused similar levels and types of color change in composite materials, with minimal effects on dental porcelain. Further *in vivo* studies are needed to establish the clinical translation of these findings.

## Figures and Tables

**Table 1:** Sample surface roughness ratios: post/pre-whitening.

	Mean Ratio + S.E.	Mean Ratio +S.E.
	Roughness After/Before: CONTROL	Roughness After/Before: TEST
**Composites**	1.43 ± 0.23Aa	1.11± 0.21Aa
**Porcelains**	1.12 ± 0.23Bb	0.07± 0.19 Bb

**Table 2:** Effects of Crest^®^ and Oral Essentials^®^ whitening strips on dL in composite over 24 hours; n=8.

Treatment	Composite-Crest^®^		Composite-Oral Essentials^®^	
Time (hours)	Mean (dL)	SD	Mean (dL)	SD
**Baseline**	0.003	0.01	0.010	0.000
**1**	0.372	0.872	−0.049	1.031
**3**	1.116	1.33	0.713	1.314
**6**	2.303	1.349	1.55	1.311
**9**	2.754	1.657	1.098	1.519
**12**	2.293	1.754	1.549	1.417
**18**	2.786	1.945	1.889	1.541
**24**	3.821	1.45	2.556	1.398

**Table 3:** Effects of Crest^®^ and Oral Essentials^®^ whitening strips on db in composite over 24 hours; n=8.

Treatment	Composite-Crest^®^		Composite-Oral Essentials^®^	
Time (hours)	Mean (db)	SD	Mean (db)	SD
**Baseline**	0	0.01	−0.003	0.009
**1**	−1.104	0.961	−0.382	0.835
**3**	−2.202	0.944	−1.174	0.567
**6**	−1.431	1.276	−1.531	0.995
**9**	−2.396	0.868	−1.89	0.898
**12**	−2.218	0.809	−1.793	0.886
**18**	−2.718	1.111	−2.071	0.669
**24**	−3.058	1.238	−2.199	1.1738

**Table 4:** Effects of Crest^®^ and Oral Essentials^®^ whitening strips on dL in porcelain at different time points (n=6).

Treatment	Porcelain-Crest^®^		Porcelain-Oral Essentials^®^	
Time (hour)	Mean (dL)	SD	Mean (dL)	SD
**Baseline**	0	0.011	0	0.011
**1**	0.1375	0.439	0.153	0.801
**3**	0.198	0.238	−0.032	0.27
**6**	0.092	0.407	0.013	0.225
**9**	0.096	0.616	−0.013	0.194
**12**	0.167	0.697	0.05	0.394
**18**	0.224	0.844	0.098	0.673
**24**	0.02	0.435	0.0223	0.17

**Table 5:** Effects of Crest^®^ and Oral Essentials^®^ whitening strips on db in porcelain at different time points (n=6).

Treatment	Porcelain-Crest^®^		Porcelain-Oral Essentials^®^	
Time (hour)	Mean (db)	SD	Mean (db)	SD
**Baseline**	0	0.011	0	0.011
**1**	−0.1	0.335	−0.084	0.575
**3**	−0.113	0.325	−0.08483	0.463
**6**	−0.0581	0.621	−0.173	0.533
**9**	−0.021	0.449	−0.101	0.44
**12**	0.0428	0.356	−0.1792	0.145
**18**	0.034	0.506	−0.1112	0.464
**24**	−0.114	0.738	−0.1795	0.789
